# Environmental Regulation, Greenwashing Behaviour, and Green Governance of High-Pollution Enterprises in China

**DOI:** 10.3390/ijerph191912539

**Published:** 2022-10-01

**Authors:** Tingfa Zhang, Huaying Qin, Weishuang Xu

**Affiliations:** 1 School of Economics and Management, Qilu Normal University, Jinan 250200, China; 2 School of Accountancy, Shandong Youth University of Political Science, Jinan 250103, China

**Keywords:** evolutionary game, greenwashing behaviour, green governance, environmental regulation, high-pollution enterprises, rent-seeking

## Abstract

This study analyses the relationship between greenwashing behaviour, a lack of government supervision, and imperfect green certification mechanisms in China. To improve green governance and greenwashing governance in light of rent-seeking behaviour between high-pollution enterprises and third-party green certification institutions (GCIs), we construct a tripartite game model for the green governance system using an evolutionary game and analyse the interaction and evolutionary trajectory between the three parties. Our results indicate that increasing local government incentives and penalties not only facilitate strengthened green governance by high-pollution enterprises that do not greenwash but also help third-party GCIs to decline to engage in rent-seeking. However, increased incentives lead to relaxed governmental supervision. In addition, the government’s incentives and penalties only meet conditions that each agent’s total incentives and penalties exceed its speculative gain, and green governance systems can be effectively prevented from resulting in unsatisfactory and unstable strategies. Moreover, the accountability that higher levels of government have to local governments effectively enables high-pollution enterprises’ refusal to greenwash and third-party GCIs’ refusal to engage in rent-seeking. Our results counsel further research on environmental regulation, green governance, and enterprises’ greenwashing, with theoretical and practical applications for both policymakers and enterprises.

## 1. Introduction

With the rapid economic progress of the last 30 years, resource and environmental issues have become a bottleneck restricting China’s economic development. China is a major polluter, and the country consistently ranks among the top worldwide in terms of energy consumption and air pollution. China’s green governance goal is to achieve peak carbon by 2030 and carbon neutrality by 2060. In the green governance process, high-pollution enterprises are the main producers of environmental pollution, the direct executive bodies of green governance, and the primary drivers of green transformation [1]. China has a battery of environmental regulations to foster green transformation and green innovation among its enterprises. In this context, consumers are increasingly beginning to prefer green consumption [2], and green products are becoming more popular [3]. According to the Ministry of Ecology and Environment’s survey results of 14 July 2020, the public’s attention to ecological environmental protection and green consumption has increased by 10% to 20% since before the COVID-19 pandemic, billions of dollars remain available to flow into environmental and social governance investment funds, and many companies are beginning to issue green bonds.

On the one hand, with the growth of the green market, society is pursuing a green economy and consumers prefer green consumption [4,5,6,7]; on the other hand, due to both insufficient penalties and information asymmetry, corporate greenwashing is a very common phenomenon [8]. Over the past 10 years, green marketing, green products, and green consumption have sparked controversy among consumers at all levels [9,10].

If enterprise greenwashing overwhelms the market, it will seriously weaken the effectiveness and execution of laws and regulations related to green products and the environment, decreasing consumer trust in formal institutions [11,12]. Moreover, enterprises’ greenwashing behaviour confuses consumers about the concepts of ‘green’ and ‘environmental protection’, causing them to wonder whether green products are legitimate [13] and to regard publicity about green products as a mere marketing strategy [14], further decreasing their willingness to purchase green products [10,15]. Therefore, the main question addressed in this study is whether environmental regulation does indeed improve companies’ green innovation to decrease greenwashing in the context of China.

To this end, numerous studies examine the greenwashing phenomenon [16,17,18] from the perspectives of institutional theory, strategic management, and information disclosure [19,20]. These studies analyse the relationship between greenwashing, environmental regulation, and information disclosure [6,8,21,22], primarily focusing on whether environmental regulation can inhibit greenwashing behaviour [7]. Studies relevant to greenwashing find that the different institutional backgrounds [19,23,24] and environmental regulations in different countries have different impacts on corporate greenwashing behaviour [24,25,26,27]. Accordingly, further research is necessary, along with analysis and comparisons of countries and regions [8]. Notably, empirical research related to green certification is extremely scarce.

In addition, most studies ignore the dilemma of Chinese local governments, that is, Chinese local governments should not only regulate environmental quality, but also maintain high-quality enterprise development and employment, and the opportunistic behaviour of corporate entities involved in green governance that greenwashing means low cost and high profit. For political reasons, local governments tend to selectively ignore the ‘greenwashing’ behaviour of high-pollution enterprises. Green certification is often provided by third-party green certification institutions (GCIs), which supplement government supervision and can in principle help to eliminate society’s ‘crisis of confidence’ in government regulation. Due to the large investment and long-term commitment that green governance would require of high-pollution enterprises, nevertheless, it is low-cost and highly profitable for them to engage in greenwashing behaviour, by which they avoid actually investing in environmental governance; therefore, high-pollution enterprises have a strong motivation to engage in greenwashing, and the behaviour of rent-seeking third-party GCIs is often difficult to curb.

We focus on high-pollution enterprises’ greenwashing behaviour and green certifications, regarding third-party GCIs as subjects of equal status to the enterprises and the government [28,29]. Using evolutionary game theory (EGT), we analyse the strategic choices and evolutionary trajectories of the three subjects of environmental regulation, the greenwashing behaviour of high-pollution enterprises, and GCI.

Our work makes a significant contribution to the literature. First, this paper enriches the literature on environmental regulation and greenwashing. To the best of our knowledge, this is the first paper to study third-party GCIs as participants in a tripartite game. Second, we use EGT, implemented in Matlab2020, to perform simulations to test the validity of the model, to conduct a sensitivity analysis of each element of the evolutionary process, and to propose suggestions and countermeasures for local governments to enhance green governance mechanisms. Finally, our study adds to the literature on greenwashing behaviour and rent-seeking by GCIs. We offer evolutionary solutions to prevent government policy failures; we intend those solutions to be constructive for policymakers, third parties, and high-pollution enterprises.

Our paper is organised as follows. Section 2 provides a detailed literature review. Section 3 provides detailed analyses of the tripartite game method, replicator dynamics, and ESSs. Section 4 provides a system simulation analysis, which verifies the validity and parameter sensitivity analysis of the proposed model. The final section presents key conclusions and the policy implications of our work.

## 2. Literature Review

### 2.1. Greenwashing Behaviour and Environmental Regulation

The research on green governance and greenwashing governance focuses on government regulation, green certification, and social supervision. Many enterprises try to lobby the government to strengthen the regulation of their competitors’ greenwashing behaviour [30]. Although measures such as environmental labels and environmental rankings can suppress such behaviour, environmental regulation is still needed to combat greenwashing [31]. Therefore, government regulation is the best approach [32,33].

To better manage the greenwashing issue, governments worldwide have formulated relevant laws and policies. For example, in 1988, the US Federal Trade Commission issued the ‘Environmental Marketing Guidelines’, which govern marketing activities, and the European Union issued the ‘EU Unfair Business Practices Directive’, which strictly supervises greenwashing advertisements. In 2007, the China’s State Environmental Protection Administration issued the ‘Measures for Disclosure of Environmental Information’, which provide new environmental protection policies, strategies, and plans. In 2010, China’s ‘Guidelines for Environmental Information Disclosure of Listed Companies’ were issued to compel the disclosure of environmental information. The 2015 ‘Environmental Protection Law’ (2015) in China imposes daily penalties to address persistent environmental violations, increasing the costs of illegal greenwashing.

Some scholars have obtained empirical evidence from the perspective of information disclosure, analysing the relationship between environmental regulation (or subsidies) and greenwashing, stakeholders, and environmental performance [19,34,35]. In addition, the abovementioned 2015 ‘Environmental Protection Law’ requires listed companies to disclose environmental information, including environmental label certification, environmental honours, environmental certification, and participation in public welfare projects related to environmental protection, ecological restoration, and environmental protection charities [36]. From the perspective of green public procurement (GPP), some scholars have deeply analysed the relationship between environmental responsibility behaviour and sustainable policy adoption and found that GPP is a key environmental policy mechanism for sustainable development and green governance [37,38].

Accordingly, many studies also concentrate on the important role of third-party environmental certifications in eliminating greenwashing [39]. Green certification systems are widely regarded as an innovative strategy to solve the problem of sustainable development [39,40,41,42,43], and green labels tend to symbolise credibility and influence [44,45]. Relevant studies show that the banking [46], oil and gas [47], hospitality [2], tourism [32], ISO14001 certification [48], and other industries believe that the third-party label certification system should be adopted, with penalties implemented, if necessary, to limit greenwashing.

In conclusion, the research on environmental regulation and enterprises’ greenwashing behaviour is insufficient. First, most studies analyse only the relationship between enterprise greenwashing and environmental regulation, failing to explore the tripartite linkages among environmental regulation, enterprises, and third-party GCIs. We want to know how high-pollution enterprises adapt their strategic behaviour to the external environment while facing pressure from environmental regulations. We also want to know how greenwashing affects environmental regulations and GCIs. Second, we consider the strategic choices related to environmental regulation, high-pollution enterprises, and green certification, which other studies seldom link to GCIs. Therefore, we use EGT to re-explore the relationship between environmental regulation, high-pollution enterprises, and green certification from the perspective of the ‘hypocrisy mechanism’ of greenwashing.

### 2.2. Application of EGT in Environmental Regulation

EGT is derived from biological evolution and combines dynamic evolutionary process with game theory, stressing dynamic equilibrium under limited rationality, that is, through replicator dynamics, agents can learn each other and ultimately achieve equilibrium.

In 1973, Smith and Price first introduced evolutionary games and evolutionary stability strategy (ESS), then another revolutionary advancement in evolutionary game theory, the replicator dynamic, was initially presented by Taylor and Jonker [49]. It was Weibull (1997) who provided a systematic and comprehensive summary of EGT and developed the core principles of the theory [50]. Increasingly, EGT is being applied to the environmental and economic field [51]. For example, using EGT, Ji et al. (2019) examined the interaction between new energy vehicle manufacturers and local governments [52]. Liu et al. (2021) elaborated on how an Ito tripartite random evolutionary game model is constructed, and the complicated interactions from three game agents were analysed [49]. Jiang et al., (2019) revealed the behavioural evolution and ESS of different participants in China by analysing the current fiscal decentralisation system [53]. For the cooperative control of heterogeneous intergovernmental smog, through an evolutionary game model, Zhang et al. (2018) analysed the dynamic evolution paths and ESS under three different conditions [54].

Additionally, some researchers have begun to apply the evolutionary game to investigate quality safety, such as agricultural product quality safety, food quality supervision and drug quality supervision, and so on. By means of EGT, establishing a patient feedback mechanism can effectively promote internet drug quality supervision [55]; furthermore, efficient and accurate new media supervision can restrain the adulteration behaviour of food companies [56,57], and government restraint mechanisms are combined with agricultural product quality safety supervision to carry out a tripartite evolutionary game [58].

Previous research of environmental regulation and rent-seeking behaviour in game theory [59,60,61,62] has laid a solid foundation for further research. Nevertheless, due to the different points of departure and research perspectives, research that includes strategy interactions among multiple players, effects such as greenwashing behaviour, GCI, and environmental regulation on evolutionary processes remains scarce. Thus, to effectively facilitate the environmental pollution control for green governance and alleviate government financial pressure, further exploration is necessary in the Chinese context.

## 3. Methodology

### 3.1. Fundamental Assumptions and Problem Description

In this paper, green governance stakeholders include local government regulators (g), high-pollution enterprises (p), and third-party GCIs, all of which are bounded rational participants. High-pollution enterprises are participant 1, third-party GCIs are participant 2, and government regulators are participant 3. Local governments have the power to constrain enterprises and compel them to invest in environmental governance, and they effectively supervise high-pollution enterprises and GCIs. Moreover, third-party GCIs are responsible for issuing environmental governance certificates, which are required before a high-pollution enterprise can sell a product.

High-pollution enterprises have two choices: to greenwash or not to greenwash. High-pollution enterprises that do not greenwash can fulfil their green responsibility to satisfy the green governance standard. High-pollution enterprises that do greenwash fail to satisfy the green governance standard. We assume that the probability of high-pollution enterprises not greenwashing is *x* and the probability of high-pollution enterprises greenwashing 1 − *x*. For a third-party GCI, *y* represents the probability that it declines to rent-seek, and 1 − *y* represents the probability that it intends to rent-seek. If *z* denotes the probability of strict supervision by the local government, then 1 − *z* is the probability of loose supervision by the local government, and we have x,y,z∈ [0,1].

According to the above analysis, we make the following basic assumptions:

- The operating profit of a high-pollution enterprise is denoted as $Q_{p}$. If the high-pollution enterprise does not greenwash, the green governance cost is denoted as $C_{p1}$; if the enterprise does greenwash, the green governance is substandard, and the environmental governance cost is denoted as $C_{p2}$ ($C_{p1}>C_{p2}$). However, if the high-pollution enterprise does not legitimately qualify for a green certificate but colludes with a third-party GCI to get it, then the GCI receives rent-seeking benefit $R_{e}$, which is the bribery cost associated with the high-pollution enterprise. Obviously, we have $R_{e}<C_{p1}-C_{p2}$. Moreover, the speculative behaviour of the high-pollution enterprise generates speculative costs $C_{p}$, mainly including the forgery of production records and false publicity.
- The inspection income of the third-party GCI is denoted as $A_{e}$. When the high-pollution enterprise fails to fulfil its green governance investment responsibilities, and if the GCI refuses to rent-seek, the GCI will decline to award the enterprise a green certificate. If the GCI intends to rent-seek, it will engage in rent-seeking behaviour with the enterprise to help the enterprise to obtain the green certificate. In addition, the speculative cost of the GCI’s intention to rent-seek is denoted as $C_{e}$, mainly including the cost of issuing false reports, falsifying testing records, issuing qualification certificates, and strengthening information security.
- When the government engages in strict supervision, high-pollution enterprises that display greenwashing behaviour and thus fail to fulfil their green governance responsibilities will be fined $F_{p}$, and environmental taxes $T_{p}$ will be levied by the government. At the same time, GCIs that intend to rent-seek are fined $F_{e}$. High-pollution enterprises that fulfil their responsibilities and eliminate environmental externalities by not greenwashing are exempt from environmental tax, and the government gives them subsidies or tax incentives $S_{p}$. Moreover, the government provides incentives for $S_{e}$ GCIs that do not rent-seek. When the government engages in loose supervision, it is impossible to obtain information about enterprises and GCIs, and the government supervision department does not have to pay incentives or impose penalties. We suppose that $C_{g}$ represents the cost of engaging in strict supervision.
- Green governance from high-pollution enterprises can provide the government with social benefits $D_{g}$. When high-pollution enterprises greenwash, thus failing to fulfil their green governance responsibilities, and reach rent-seeking agreements with GCIs, the government needs to eliminate environmental externalities, and the required environmental governance cost is denoted as $E_{g}$. When the local government adopts a loose supervision strategy that results in lack of supervision, it is subjected to the accountability and administrative penalty $F_{g}$ from the higher-level government department. Generally, we have $F_{g}>C_{g}$.

### 3.2. Analysis of the ESS of Tripartite Game Subject

According to the above assumptions, when high-pollution enterprises, local governments, and GCIs participate in different combinations of strategies, the payoff matrix appears as Table 1.

#### 3.2.1. Analysis of the ESS of High-Pollution Enterprises

Let $U_{11}$ and $U_{12}$ represent the expected returns of high-pollution enterprises without greenwashing and with greenwashing, respectively, and let ${\bar{U}}_{1}$ represent the average expected return.

(1) $$\begin{array}{cc} U_{11} & =yz[Q_{p}-C_{p1}+S_{P}]+y(1-z)[Q_{p}-C_{p1}]+(1-y)z[Q_{p}-C_{p1}+S_{P}]\\ & +(1-y)(1-z)[Q_{p}-C_{p1}] \end{array}$$

(2) $$\begin{array}{cc} U_{12} & =yz[-C_{p2}-C_{p}-F_{p}-T_{p}]+(1-z)y[-C_{p2}-C_{p}-T_{p}]\\ & +(1-y)z[Q_{p}-C_{p2}-C_{p}-R_{e}-F_{p}-T_{p}]\\ & +(1-z)(1-y)[Q_{p}-C_{p2}-C_{p}-R_{e}-T_{p}] \end{array}$$

(3) $$\bar{U}=xU_{11}+(1-x)U_{12}$$

Consequently, high-pollution enterprises have the following replication dynamic equation:

(4) $$\begin{array}{cc} F(x) & =x(U_{11}-{\bar{U}}_{1})=x(x-1)[(C_{p1}-C_{p2}-C_{p}-R_{e}-T_{p})\\ & -y(Q_{p}-R_{e})-z(F_{p}+S_{p})] \end{array}$$

Then, F(x) has the following first derivative:

(5) $$d(F(x))/dx=(2x-1)[(C_{p1}-C_{p2}-C_{p}-R_{e}-T_{p})-y(Q_{p}-R_{e})-z(F_{p}+S_{p})]$$

Let $G(y,z)=(C_{p1}-C_{p2}-C_{p}-R_{e}-T_{p})-y(Q_{p}-R_{e})-z(F_{p}+S_{p})$.

We know from the stability theorem of the replication dynamic equation that when F(x)=0 and $F^{\prime }(x)<0$ are satisfied, *x* is the ESS of the high-pollution enterprises.

Let F(x)=0; hence, we have x=1, x=0, $y^{*}=[C_{p1}-C_{p2}-C_{p}-R_{e}-T_{p})-z(F_{p}+S_{p})]/(Q_{p}-R_{e})$.

Due to ∂G(y,z)/∂y<0, G(y,z) with respect to y is a decreasing function.

Therefore, when $y=y^{*}$ is satisfied, we have F(x)=0, and then the selection probability of the high-pollution enterprises does not change with time. When $0<y<y^{*}$, we have $F^{\prime }(0)<0$ and $F^{\prime }(1)>0$, and then x=0 represents the ESS for the high-pollution enterprises. Similarly, when $y>y^{*}$ is satisfied, x=1 represents the ESS for the high-pollution enterprises.

**Figure 1:**
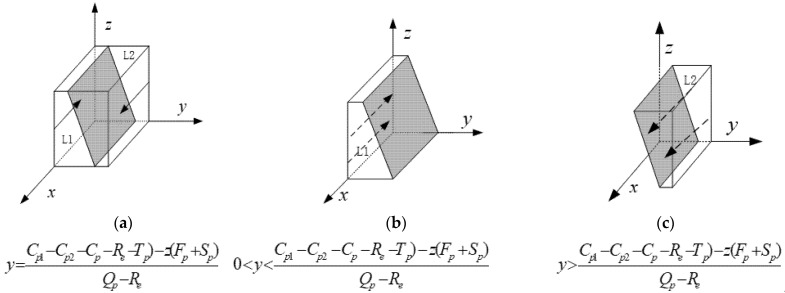
The phase diagram of high-pollution enterprises’ replication dynamics.

The phase diagram of the high-pollution enterprises is depicted in Figure 1.

Figure 1 shows that the probability *L*1 that the green governance of high-pollution enterprises fails to meet the standard is the volume *V_L_*_1_ (i.e., the double integral of the cross-sectional function on the X-Z plane); the probability *L*2 that the green governance meets the standard is the volume *V_L_*_2_.

(6) $$\begin{array}{cc} V_{L2} & =1-V_{L1}=1-\int_{0}^{1}\int_{0}^{1}\frac{C_{p1}-C_{p2}-C_{p}-R_{e}-T_{p})-z(F_{p}+S_{p})}{Q_{p}-R_{e}}dzdx\\ & =\frac{2Q_{p}-2C_{p1}+2C_{p2}+2C_{p}+2T_{p}+F_{p}+S_{p}}{2(Q_{p}-R_{e})} \end{array}$$

**Proposition** **1.** *The probability of the high-pollution enterprises meeting the green governance standards has a positive correlation with the government’s incentive–penalty, environmental taxes, rent-seeking costs, speculation costs, and operating profits but is negatively related to the cost saved by high-pollution enterprises that fail to meet green governance standards.*

**Proof** **of** **Proposition** **1.** According to Equation (6), we can obtain the first-order partial derivatives as follows.

$$\partial V_{L2}/\partial (F_{p}+S_{p})>0,\partial V_{L2}/\partial T_{p}>0,\partial V_{L2}/\partial R_{e}>0,\partial V_{L2}/\partial C_{p}>0,$$

$\partial V_{L2}/\partial Q_{p}>0$, $\partial V_{L2}/\partial (C_{p1}-C_{p2})<0$. Therefore, if $F_{p}+S_{p}$, $T_{p}$, $R_{e}$, $C_{p}$, and $R_{p}$ increase, or if $\left(C_{p1}-C_{p2}\right)$ decreases, the probability of the high-pollution enterprises meeting the green governance standards increases. □

**Proposition** **2.** *During the evolution process, when the probability of the third-party GCI declining to rent-seek and the probability of strict supervision by the local government increase, the probability of high-pollution enterprises fulfilling their green governance responsibilities increases.*

**Proof** **of** **Proposition** **2.** It can be seen from the ESS of the above high-pollution enterprises that when $y<y^{*}$, the ESS for the high-pollution enterprises is x=0; when $y>y^{*}$, x=1, then the ESS for the high-pollution enterprises is x=1. Similarly, when $z<z^{*}$, the ESS for the high-pollution enterprises is x=0; when $z>z^{*}$, the ESS for the high-pollution enterprises is x=1. Therefore, when the values of *z* and *y* gradually increase, the value of *x* in the stabilisation strategy of the high-pollution enterprises increases from *x* = 0 (environmental non-compliance) until *x* = 1 (environmental compliance). □

#### 3.2.2. Strategy Stability Analysis of Third-Party GCIs

Let $U_{21}$ and $U_{22}$ represent the expected returns of the third-party GCIs for declining to rent-seek and intending to rent-seek, respectively, and let ${\bar{U}}_{2}$ represent the average expected return. Then, we have

(7) $$U_{21}=x[z(A_{e}+S_{e})+(1-z)A_{e}]+(1-x)[z(A_{e}+S_{e})+(1-z)A_{e}]$$

(8) $$U_{22}=x[z(A_{e}-C_{e}-F_{e})+(1-z)(A_{e}-C_{e})]+(1-x)[A_{e}-C_{e}+R_{e}-zF_{e}]$$

(9) $${\bar{U}}_{2}=yU_{21}+(1-y)U_{22}$$

Hence, the third-party GCIs have the following replicator dynamics equation:

(10) $$F(y)=dy/dx=y(U_{21}-{\bar{U}}_{2})=y(y-1)[(1-x)R_{e}-z(F_{e}+S_{e})-C_{e}]$$

Then, F(y) has the following first derivative:

$$d(F(y))/dy=(2y-1)[(1-x)R_{e}-z(F_{e}+S_{e})-C_{e}].\;\mathrm{Let}\;L(x,z)=(1-x)R_{e}-z(F_{e}+S_{e})-C_{e}.$$

We find from the replication dynamic equation’s stability theorem that if F(y)=0 and $F^{\prime }(y)<0$ are satisfied, *y* signifies the ESS of the third-party GCI. Let F(y)=0, and then, we obtain y=0, y=1, $z^{*}=[(1-x)R_{e}-C_{e}]/(F_{e}+S_{e})$. Due to ∂L(x,z)/∂z<0, L(x,z) with respect to z is a decreasing function. Therefore, when $z=z^{*}$ is satisfied, we have F(y)=0, and then, the selection probability of the GCI does not vary with time. When $0<z<z^{*}$, we have $F^{\prime }(0)<0$ and $F^{\prime }(1)>0$, and then, y=0 represents the ESS of the GCI.

Similarly, when $z>z^{*}$ is satisfied, y=1 denotes the ESS of the third-party GCI.

**Figure 2:**
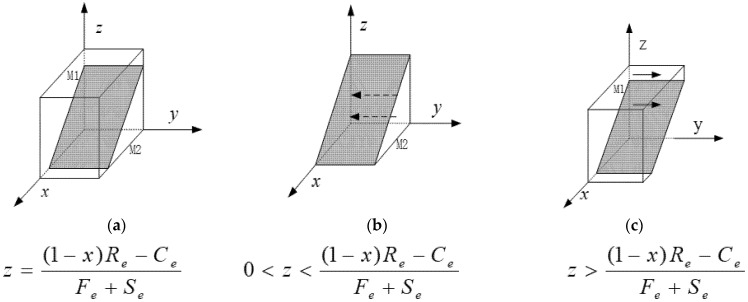
The phase diagram of the third-party GCI’s replication dynamics.

The phase diagram for the third-party GCI is described in Figure 2.

Figure 2 indicates that the probability *M*1 of the GCI declining to rent-seek is the volume *V_M_*_1_, and the probability *M*2 of the GCI intending to rent-seek is the volume *V_M_*_2_. The section passes through the point ($(R_{e}-C_{e})/R_{e}$, 0, 0), whose projection plane is *x*–*y*, and then, we have

(11) $$V_{M1}=1-V_{M2}=1-\int_{0}^{1}\int_{0}^{(R_{e}-C_{e})/R_{e}}\frac{(1-x)R_{e}-C_{e}}{F_{e}+S_{e}}dxdy=1-\frac{{\left(R_{e}-C_{e}\right)}^{2}}{2(F_{e}+S_{e})R_{e}}$$

**Proposition** **3.** *The probability that the third-party GCI declines to rent-seek has a positive correlation with the government’s incentive–penalty system and the third party’s speculative cost but is negatively correlated with the third-party’s rent-seeking income.*

**Proof** **of** **Proposition** **3.** According to Equation (11), we can obtain the first-order partial derivatives as follows.

$\partial V_{M1}/\partial F_{e}>0$, $\partial V_{M1}/\partial S_{e}>0$, $\partial V_{M1}/\partial C_{e}>0$, $\partial V_{M1}/\partial R_{e}<0$. Therefore, the increase in $F_{e}$, $S_{e}$, $C_{e}$, or the decrease in $R_{e}$ can encourage the third-party GCI to decline to rent-seek. □

**Proposition** **4.** *During the evolution process, when the probabilities of strict government supervision and the high-pollution enterprises meeting green governance standards increase, the probability of the GCIs declining to rent-seek increases accordingly.*

**Proof** **of** **Proposition** **4.** It is clear that because L(x,z) is also a decreasing function of x, this proposition can be proved in a similar manner to Proof 2. □

#### 3.2.3. Analysis of the ESS of Local Government Regulators

Let $U_{31}$ and $U_{32}$ denote the expected returns of strict supervision and loose supervision by the local government, respectively, and let ${\bar{U}}_{3}$ represent the average expected return.

(12) $$\begin{array}{cc} U_{31} & =-C_{g}-xS_{p}+xD_{g}-yS_{e}+(1-x)F_{p}+(1-y)F_{e}\\ & +(1-y)(1-x)T_{p}-(1-y)(1-x)E_{g} \end{array}$$

(13) $$\begin{array}{cc} U_{32} & =x[yD_{g}+(1-y)D_{g}]+(1-x)[y^{*}0+(1-y)(-E_{g}-F_{g})]\\ & =xD_{g}-(x-1)(y-1)(E_{g}+F_{g}) \end{array}$$

(14) $${\bar{U}}_{3}=zU_{31}+(1-z)U_{32}$$

Then, we have the government’s replication dynamics equation as follows:

(15) $$\begin{array}{cc} F(z) & =z(U_{31}-{\bar{U}}_{3})=z(z-1)[(C_{g}-F_{e}-F_{p}-T_{P}-F_{g})+x(S_{p}+F_{p}+T_{P}+F_{g})\\ & +y(S_{e}+F_{e}+T_{P}+F_{g})-xy(T_{P}+F_{g})] \end{array}$$

Accordingly, F(z) has the following first-order derivative:

$$\begin{array}{cc} d(F(z))/dz & =(2z-1)[(C_{g}-F_{e}-F_{p}-T_{P}-F_{g})+x(S_{p}+F_{p}+T_{P}+F_{g})\\ & +y(S_{e}+F_{e}+T_{P}+F_{g})-xy(T_{P}+F_{g})] \end{array}$$

Let $H(x,y)=(C_{g}-F_{e}-F_{p}-T_{P}-F_{g})+x(S_{p}+F_{p}+T_{P}+F_{g})+y(S_{e}+F_{e}+T_{P}+F_{g})-xy(T_{P}+F_{g})$.

Similarly, when F(z)=0 and $F^{\prime }(z)<0$ are satisfied, z is the local government’s ESS.

Let F(z)=0, and then, we obtain z=0, z=1, and

$$y^{**}=\frac{\left(F_{e}+F_{p}+T_{P}+F_{g}-C_{g})-x(S_{p}+F_{p}+T_{P}+F_{g}\right)}{(S_{e}+F_{e}+T_{P}+F_{g})-xT_{P}-xF_{g}}$$

Due to ∂H(x,y)/∂y>0, the function H(x,y) of y is an increasing function. Hence, if $y=y^{**}$ is met, we have H(x,y)=0, and the selection probability of the local government does not vary with time. When $0<y<y^{**}$, we can obtain $F^{\prime }(0)>0$ and $F^{\prime }(1)<0$, then z=1 represents the ESS of the local government. Similarly, while $y>y^{**}$ is satisfied, z=0 denotes the ESS of the local government.

**Figure 3:**
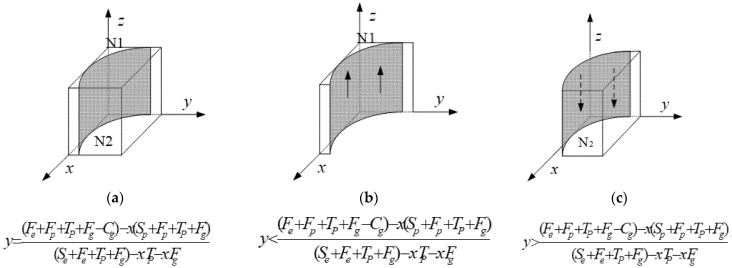
Phase diagram of the local government’s replication dynamics.

Figure 3 presents the phase diagram of the local government.

**Figure 4:**
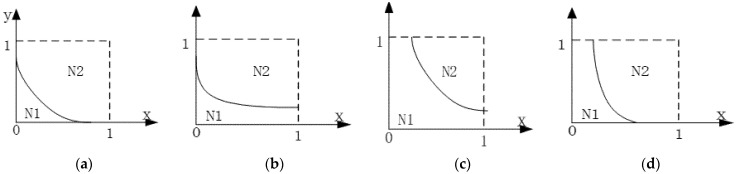
The intersection of the surface with the graph in the plane X-O-Y.

Figure 3 indicates that the volumes of *N*1 and *N*2 are equal to the probability of strict supervision and loose supervision by the local government, respectively. The curved surface H(x,y) is parallel to the *z* axis. We discuss the intersection of the surface in the *x–y* plane. There are four possible situations, as shown in Figure 4, and so, the intersection may be expressed as follows, from which we can observe that the change of point coordinates is in accord with *V_N_*_1_.

Without loss of generality, we discuss the situation in Figure 4d.

(16) $$\mathrm{Let}\;y^{*}=\frac{F_{e}+F_{p}+T_{P}+F_{g}-C_{g}}{S_{e}+F_{e}+T_{P}+F_{g}},x^{*}=\frac{F_{e}+F_{p}+T_{P}+F_{g}-C_{g}}{S_{p}+F_{p}+T_{P}+F_{g}}$$

**Proposition** **5.** *Under certain conditions, the probability of strict supervision by the local government has a positive correlation with environmental taxes, penalties imposed on high-pollution enterprises, penalties imposed on GCIs, and administrative penalties imposed on local governments by higher levels of government, but a negative correlation with incentives that are given to high-pollution enterprises and GCIs by the local government.*

**Proof** **of** **Proposition** **5.** Due to $y^{*}=\frac{F_{e}+F_{p}+T_{P}+F_{g}-C_{g}}{S_{e}+F_{e}+T_{P}+F_{g}}=1+\frac{F_{p}-S_{e}-C_{g}}{S_{e}+F_{e}+T_{P}+F_{g}}$, it is evident that when $F_{p}-S_{e}-C_{g}<0$, $y^{*}$ and $F_{e}$ are positively correlated. In addition, $x^{*}$ is positively correlated with $F_{e}$, according to Equation (16), and $V_{N1}$ is also positively correlated with $F_{e}$. Due to $x^{*}=\frac{F_{e}+F_{p}+T_{P}+F_{g}-C_{g}}{S_{p}+F_{p}+T_{P}+F_{g}}=1+\frac{F_{e}-S_{p}-C_{g}}{S_{p}+F_{p}+T_{P}+F_{g}}$, similarly, when $F_{p}-S_{p}-C_{g}<0$, $x^{*}$ and $y^{*}$ are positively correlated with $F_{p}$. Subsequently, $V_{N1}$ is positively correlated with $F_{p}$. When $F_{p}-S_{e}-C_{g}<0$, $F_{p}-S_{p}-C_{g}<0$, $x^{*}$ and $y^{*}$ are positively correlated with $F_{g}$. Other conclusions can be similarly proved. □

**Proposition** **6.** *In the evolution process, when the probabilities of high-pollution enterprises meeting green governance standards and third-party GCIs declining to rent-seek increase, the probability of strict supervision by the local government decreases.*

**Proof** **of** **Proposition** **6.** Due to $H(x,y)=(C_{g}-F_{e}-F_{p}-T_{P}-F_{g})+x(S_{p}+F_{p}+T_{P}+F_{g})+y(S_{e}+F_{e}+T_{P}+F_{g})-xy(T_{P}+F_{g})$, it is obvious that the correlation between H(x,y) and x,y is consistent. Therefore, with the increase in *x* and *y*, it can be seen from Figure 3 that the probability of strict government regulation decreases from *z* = 1 to *z* = 0, that is, *z* will decrease with the increase in *x* and *y*. This shows that when the probabilities of high-pollution enterprises meeting the green governance standards or of GCIs declining to rent-seek increase, the probability of strict supervision by the local government decreases, and the local government is prone to provide little supervision. □

### 3.3. Stability Analysis of the Green Governance System

The dynamic replication equations of the green governance system are given by Equations (4), (10) and (15).

(17) $$\left\{\begin{array}{c} F(x)=x(x-1)G(y,z)\\ F(y)=y(y-1)L(x,z)\\ F(z)=z(z-1)H(x,y) \end{array}\right.$$

Let F(x)=0, F(y)=0, F(z)=0, and then, the 14-system equilibrium point is obtained. Specifically, there are eight pure-strategy equilibrium solutions, i.e., *M*_1_ (0, 0, 0), *M*_2_ (0, 1, 0), *M*_3_ (0, 0, 1), *M*_4_ (0, 1, 1), *M*_5_ (1, 0, 0), *M*_6_ (1, 1, 0), *M*_7_ (1, 0, 1), and *M*_8_ (1, 1, 1); and six mixed-strategy equilibrium solutions, i.e.,

$$\begin{array}{c} M_{9}(-(C_{g}-F_{p}+S_{e})/(F_{p}+S_{p}),1,(-(C_{p}-C_{p1}+C_{p2}+Q_{p}+T_{p}))/(F_{p}+S_{p}))\\ M_{10}(1,(-(C_{g}-F_{e}+S_{p}))/(F_{e}+S_{e}),-C_{e}/(F_{e}+S_{e}))\\ M_{11}((F_{p}-C_{g}+F_{e}+T_{p}+F_{g})/(F_{p}+S_{p}+T_{p}+F_{g}),0,-(R_{e}+C_{p}-C_{p1}+C_{p2}+T_{p})/(F_{p}+S_{p}))\\ M_{12}((R_{e}-C_{e})/R_{e},(R_{e}+C_{p}-C_{p1}+C_{p2}+T_{p})/(R_{e}-Q_{p}),0)\\ M_{13}(-(C_{e}-R_{e}+F_{e}+S_{s})/R_{e},(R_{e}+C_{p}-C_{p1}+C_{p2}+F_{p}+T_{p})/(R_{e}-Q_{p}),1)\\ M_{14}(0,(F_{p}-C_{g}+F_{e}+T_{p}+F_{g})/(F_{e}+S_{e}+T_{p}+F_{g}),(R_{e}-C_{e})/(F_{e}+S_{e})) \end{array}$$

These six mixed equilibrium solutions are only established under certain conditions, and because mixed strategies are not ESS [42], only the eight pure strategy equilibrium points need to be discussed.

Noteworthily, the equilibrium solutions are not all ESS. In term of Lyapunov’s first method, if all of the eigenvalues of the Jacobian matrix are negative, the equilibrium solution is the ESS; if the signs of all the eigenvalues of the Jacobian matrix are determined and there are positive eigenvalues, the equilibrium solution is the unstable point.

Then, the Jacobian matrix *J* of the green governance system is derived as follows:

$$J=\left(\begin{array}{ccc} \left(2x-1)G(y,z\right) & x(x-1)(R_{e}-Q_{p}) & x(x-1)(-F_{p}-S_{p})\\ y(y-1)(-R_{e}) & \left(2y-1)L(x,z\right) & y(y-1)(-F_{e}-S_{e})\\ z(z-1)J(y) & z(z-1)S(x) & \left(2z-1)H(x,y\right) \end{array}\right)$$

where $G(y,z)=-y(Q_{p}-R_{e})-z(F_{p}+S_{p})+(C_{p1}-C_{p2}-C_{p}-R_{e}-T_{p})$, $L(x,z)=(1-x)R_{e}-z(F_{e}+S_{e})-C_{e}$, $J(y)=(S_{p}+F_{p}+T_{P}+F_{g})-y(T_{P}+F_{g})$, $S(x)=(S_{e}+F_{e}+T_{P}+F_{g})-x(T_{P}+F_{g})$, $H(x,y)=(C_{g}-F_{e}-F_{p}-T_{P}-F_{g})+x(S_{p}+F_{p}+T_{P}+F_{g})+y(S_{e}+F_{e}+T_{P}+F_{g})-xy(T_{P}+F_{g})$.

Table 2 shows the Jacobian matrix eigenvalues.

**Proposition** **7.** *When $C_{e}-R_{e}+F_{e}+S_{e}<0$ and $C_{p}-C_{p1}+C_{p2}+F_{p}+T_{p}+S_{p}+R_{e}<0$, namely, $F_{e}+S_{e}<R_{e}-C_{e}$ and $F_{p}+T_{p}+S_{p}+R_{e}<C_{p1}-C_{p2}-C_{p}$, the replicator dynamic system has two stable points, that is, M_3_ (0, 0, 1) and M_6_ (1, 1, 0).*

**Proof** **of** **Proposition** **7.** When the condition of Proposition 7 is satisfied, it can be concluded from Table 2 that the Jacobian matrix eigenvalues corresponding to the equilibrium points *M*_3_ (0, 0, 1) and *M*_6_ (1, 1, 0) are all negative, which illustrates that *C* (0, 0, 1) and *F* (1, 1, 0) are two stable points. □

Proposition 7 shows that when the local government’s incentives, environmental taxes, and penalties (i.e., $F_{e}+S_{e}$ and $F_{p}+T_{p}+S_{p}$) for the high-pollution enterprises and GCIs are relatively small, or when the high-pollution enterprises greenwash and do not fulfil their green governance responsibilities, the speculative returns (i.e., $C_{p1}-C_{p2}-C_{p}$) are very high, and at the same time, the rent-seeking income ($R_{e}$) of the GCI is also high. In that situation, the strategy portfolio eventually evolves into two stable strategies (substandard green governance, intention to rent-seek, and strict supervision; and standard green governance, refusal to rent-seek, and loose supervision), although the initial points of the three-party strategy selection are different. To prevent the appearance of the former strategy, the local government must set its penalties or incentives at a sufficiently high level.

**Proposition** **8.** *When $C_{e}-R_{e}+F_{e}+S_{e}>0$ and $C_{p}-C_{p1}+C_{p2}+F_{p}+T_{p}+S_{p}+R_{e}>0$, namely, $F_{e}+S_{e}>R_{e}-C_{e}$ and $F_{p}+T_{p}+S_{p}+R_{e}>C_{p1}-C_{p2}-C_{p}$, there is only one stable point M_6_ (1, 1, 0) for a replicator dynamic system.*

**Proof** **of** **Proposition** **8.** According to Table 2, when the above conditions are met, the eigenvalues of the Jacobian matrix in accordance with the equilibrium point *M*_6_ (1, 1, 0) are completely negative, which illustrates that *M*_6_ (1, 1, 0) are unique stable points. □

Proposition 8 illustrates that only when the sum (i.e., $F_{p}+T_{p}+S_{p}+R_{e}$ and $F_{e}+S_{e}$) of the local government’s incentives, environmental taxes, and penalties for high-pollution enterprises and GCIs is higher than their corresponding speculative gains (i.e., $C_{p1}-C_{p2}-C_{p}$ and $R_{e}-C_{e}$) can the stable strategy (substandard green governance, intention to rent-seek, and strict supervision) be effectively prevented in the green governance system. Moreover, the changes in the operating profits of the high-pollution enterprises, the cost of strict government supervision, and the administrative penalties imposed on local governments by higher levels of government cannot transform the evolutionary results.

## 4. System Simulation Results and Analysis

The above evolutionary stability analysis is validated by assigning numerical values. Matlab2020b was used for numerical simulation.

### 4.1. The Effect of Different Initial Parameters on the Evolutionary Process

Two sets of values that satisfy Propositions 7 and 8 are chosen, and the two sets of values are expressed as follows: array 1: $T_{p}=10$, $Q_{p}=150$, $C_{p}=10$, $R_{e}=35$, $C_{p1}-C_{p2}=100$, $F_{p}=20$, $S_{p}=15$, $C_{e}=10$, $F_{e}=12$, $S_{e}=10$, $C_{g}=15$, $F_{g}=40$; array 2: $T_{p}=10$, $C_{p}=10$, $Q_{p}=150$, $C_{p1}-C_{p2}=70$, $R_{e}=30$, $F_{p}=30$, $S_{p}=20$, $C_{e}=10$, $F_{e}=20$, $S_{e}=15$, $C_{g}=15$, $F_{g}=40$.

**Figure 5:**
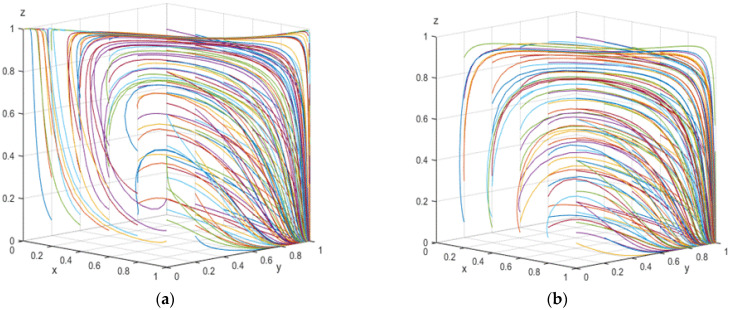
(**a**) The result of evolutions of array 1. (**b**) The result of evolutions of array 2.

For the above two sets of values, the replication dynamics system starts from different initial parameters and evolves over time for simulation. A summary of the results can be found in Figure 5.

Figure 5a shows that under the condition of satisfying Proposition 7, there are two evolutionary stable points *C* (0, 0, 1), *F* (1, 1, 0) in the three-party evolutionary game system (substandard green governance, intention to rent-seek, and strict supervision; and standard green governance, refusal to rent-seek, and loose supervision). It can be seen from Figure 5b that under the condition of satisfying Proposition 8, *F* (1, 1, 0) is a unique stable point, and the simulation analysis is consistent with the conclusions of Propositions 7 and 8.

### 4.2. Sensitivity Analysis and Discussion

We chose a second set of values that satisfy only one stable point to analyse the effects of $T_{p}$, $F_{p}$, $Q_{p}$, $R_{e}$, $F_{e}$, $S_{e}$, $S_{p}$, and $F_{g}$ on the evolutionary process and results. We assume that the initial decision-making probability among the high-pollution enterprise, the GCI, and the local government is set to (0.2, 0.2, 0.2), and the replication dynamics system evolves for simulation.

**Figure 6:**
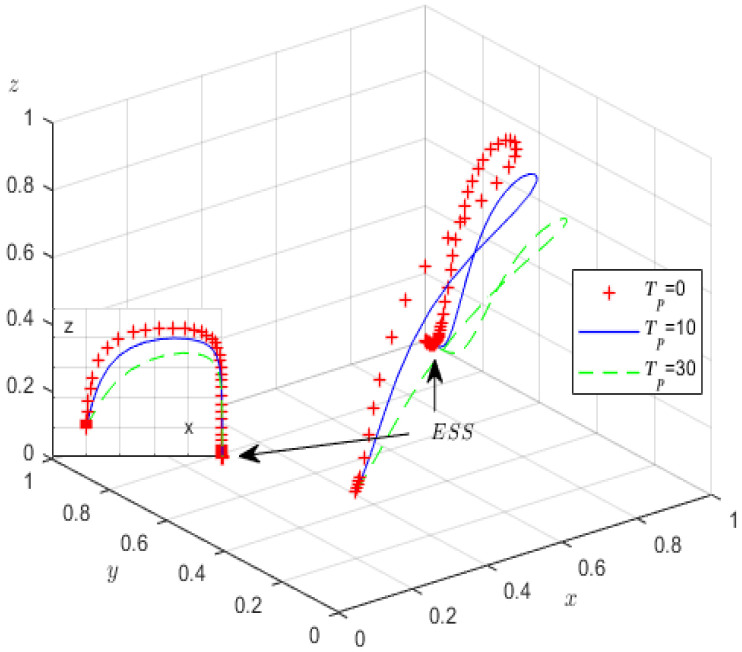
The influence of *T_p_* on evolutionary processes.

**Figure 7:**
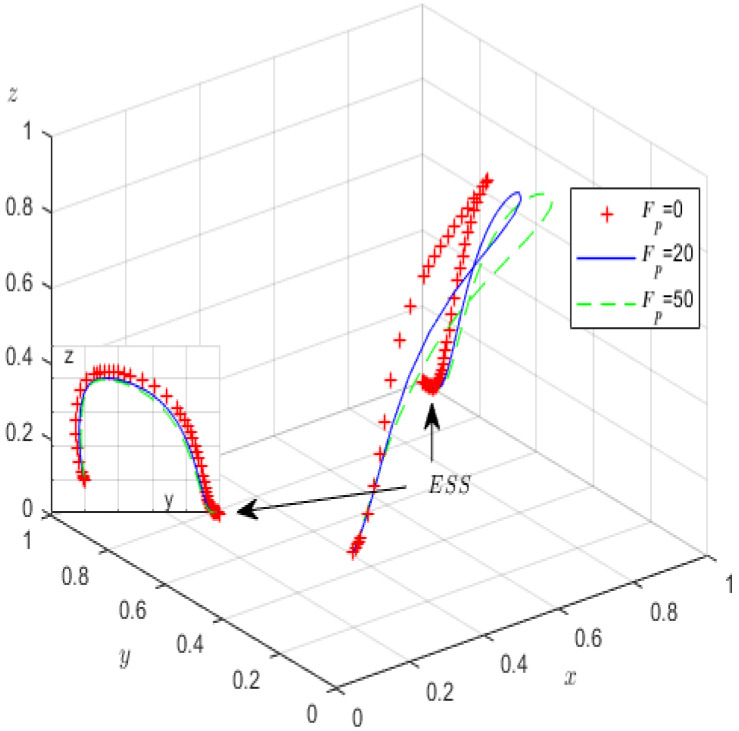
The influence of *F_p_* on evolutionary processes.

First, we analysed how environmental taxes and penalties imposed on high-pollution enterprises influence the evolutionary game process and outcomes. Therefore, $T_{p}$ is assigned values of 0, 10, 30, $F_{p}$ is assigned values of 0, 20, 50, and the other parameters are fixed, as shown in Figure 6 and Figure 7.

Figure 6 shows that increasing environmental taxes significantly accelerates the evolution of high-pollution enterprises. When environmental taxes increase, the probability of high-pollution enterprises meeting green governance standards increases, but the probability of strict supervision by the local government decreases. Therefore, increased environmental taxes help high-pollution enterprises to fulfil their green governance responsibilities. Nevertheless, the probability of strict government regulation decreases, primarily because reliance on environmental taxes appears to decrease the probability of strict supervision. From Figure 7, it can be concluded that increasing government penalties on high-pollution enterprises can accelerate the evolution of high-pollution enterprises. With increasing local government penalties on high-pollution enterprises, the probability of strict supervision by the local government gradually decreases.

**Figure 8:**
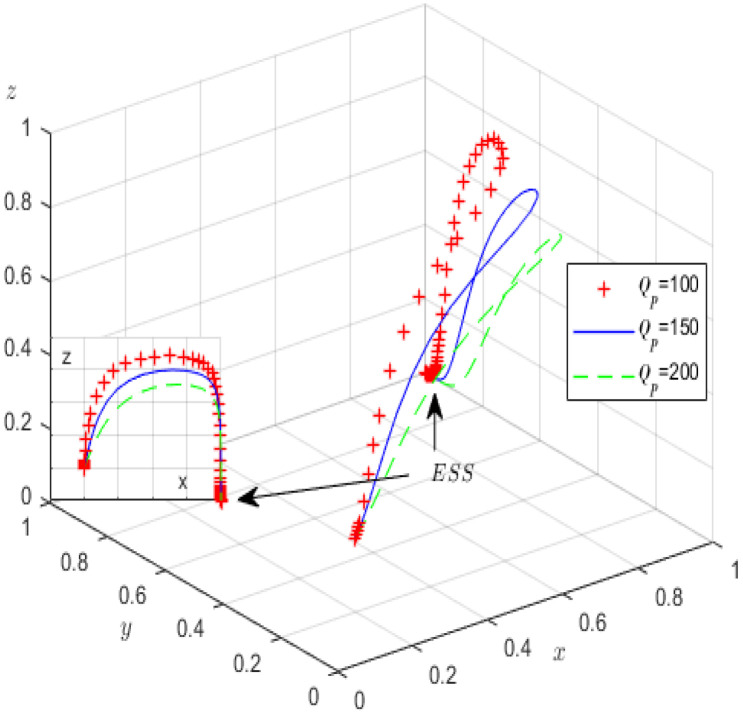
The influence of *Q_p_* on evolutionary processes.

**Figure 9:**
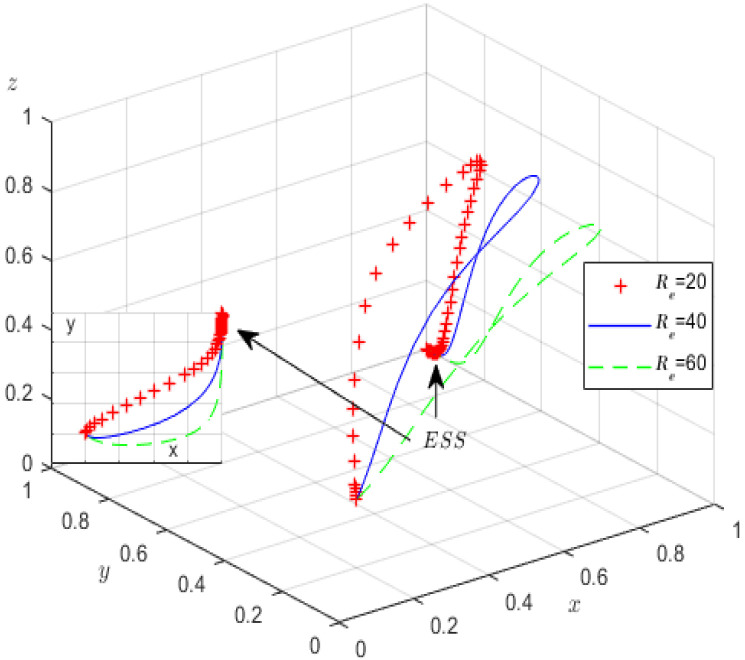
The influence of *R_e_* on evolutionary processes.

Second, in the case of fixing the other parameters except oneself, the impact of $Q_{p}$ and $R_{e}$ on the evolutionary game processes and results is analysed. $Q_{p}$ is assigned values of 100, 150, 200, and $R_{e}$ is correspondingly assigned values of 20, 40, 60. Figure 8 and Figure 9 provide the simulation processes.

Similar to Figure 6, during the evolution process of the tripartite system in Figure 8, increasing the profits of high-pollution enterprises accelerates their evolution. We find that although the probability of high-pollution enterprises meeting the green governance standards increases, the probability of strict supervision by the local government decreases. We can see from Figure 9 that during the evolutionary process, with increased rent-seeking costs, the probability of high-pollution enterprises actively fulfilling their environmental governance responsibilities significantly increases; conversely, the probability of third-party GCIs refusing rent-seeking significantly decreases. Obviously, local governments can adopt various measures, such as expanding the reputational influence of enterprises, increasing their media disclosure capabilities, and cultivating consumers’ environmental awareness, all of which can increase the high-pollution enterprises’ rent-seeking costs and encourage them to perform green governance; however, the increase in rent-seeking income makes GCIs more likely to rent-seek.

**Figure 10:**
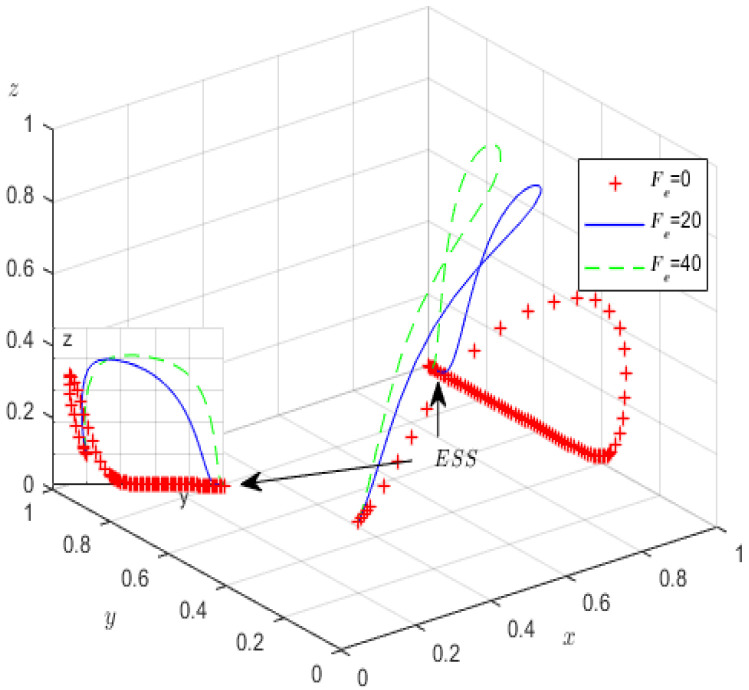
The influence of *F_e_* on evolutionary processes.

**Figure 11:**
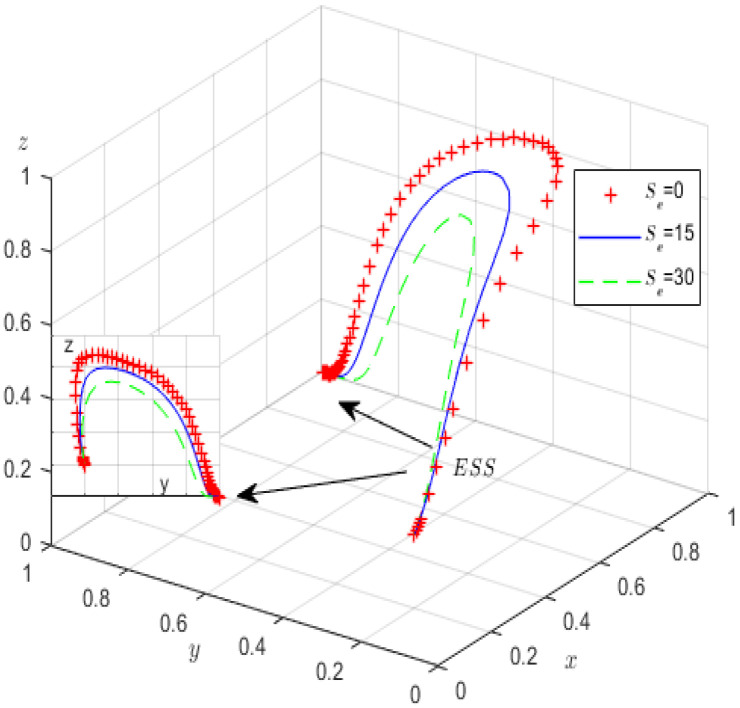
The influence of *S_e_* on evolutionary processes.

Next, to analyse how parameters $F_{e}$ and $S_{e}$ influence the evolutionary game process and outcomes, we assign $F_{e}$ values of 0, 20, 40, and $S_{e}$ values of 0, 15, 30, and the other parameters are fixed. Figure 10 and Figure 11 illustrate the results of the simulation.

We see from Figure 10 that before the green governance of high-pollution enterprises stabilises at 1, if the local government’s penalties for GCIs increase, then the probabilities of strict supervision by the local government and GCIs declining to rent-seek increase, but it is worth noting that after the green governance strategy of high-pollution enterprises stabilises at 1, the probability of strict supervision by the government gradually decreases and stabilises at 0. Figure 11 illustrates that in the course of evolution, reduction in strict government oversight is likely if local government incentives increase for third-party GCIs, perhaps due to higher subsidies inhibiting the local governments’ expected income. Therefore, the local governments’ incentives and penalties should be controlled, at least within a certain range.

**Figure 12:**
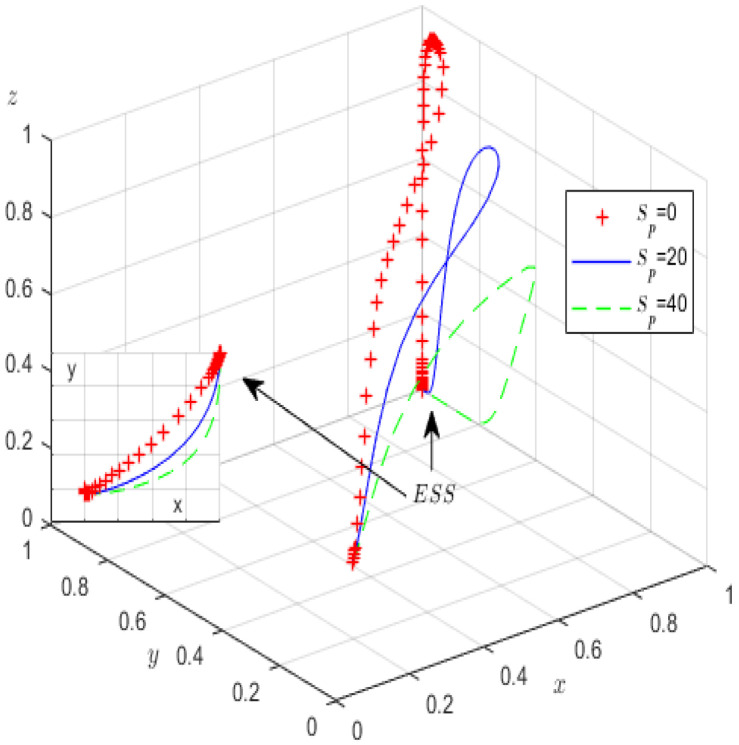
The influence of *S_p_* on evolutionary processes.

**Figure 13:**
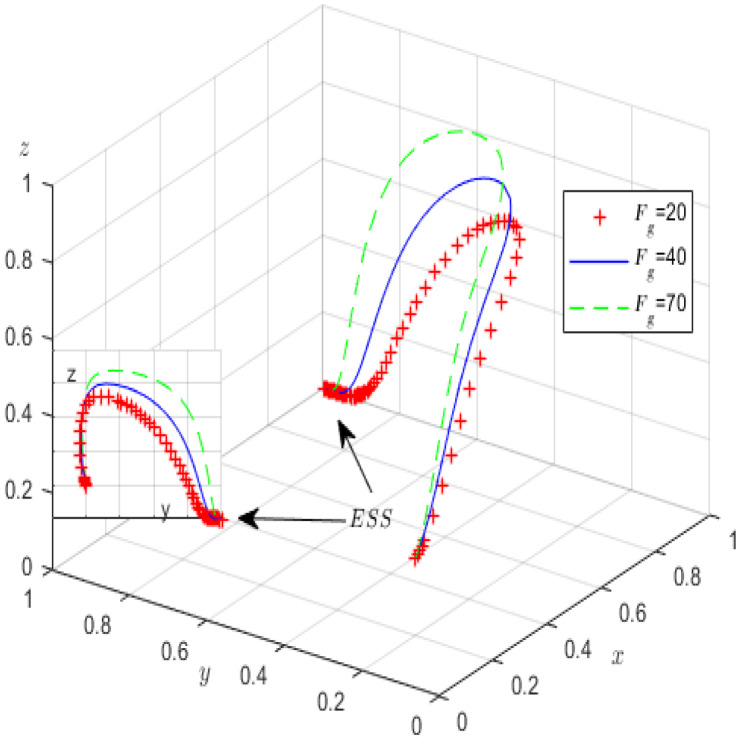
The influence of *F_g_* on evolutionary processes.

Furthermore, to analyse how parameters $S_{p}$ and $F_{g}$ affect the evolutionary game process and results, $S_{p}$ is allocated values of 0, 20, 40, and $F_{g}$ is allocated values of 20, 40, and 60. In Figure 12 and Figure 13, we provide the simulation results.

As is shown in Figure 12, in the evolution process, when local governments increase the incentives for high-pollution enterprises, the probability of high-pollution enterprises actively fulfilling their green governance responsibilities significantly increases, but the probability of third-party GCIs refusing to rent-seek significantly decreases, further illustrating that although local government incentives for high-pollution enterprises can promote green governance, they are not conducive to the performance of the regulatory authorities’ duties. Figure 13 shows that before the environmental governance probability of high-pollution enterprises stabilises at 1, when higher levels of government impose higher administrative penalties on local governments, there is a greater probability that local governments strictly supervise compliance with regulations and third-party GCIs decline to rent-seek. This shows that severe penalties imposed by higher levels of government encourage local governments to strictly supervise enterprises and enforce a high rate of compliance, further enhancing the robustness of corporate environmental governance.

### 4.3. The Influence of the Initial Probability of Three Agents on the Evolutionary Process

**Figure 14:**
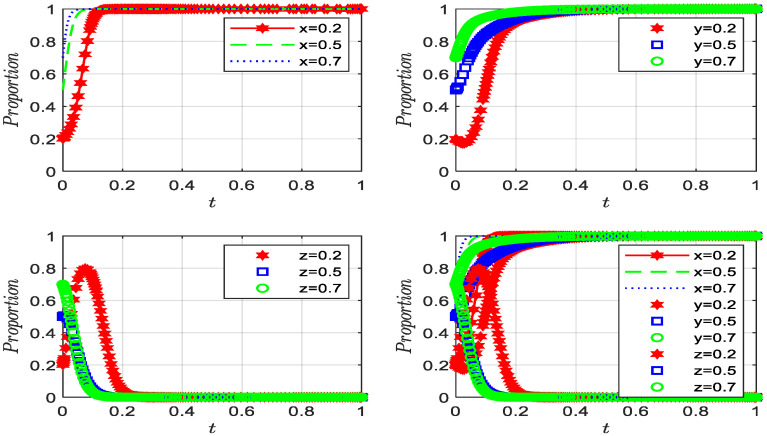
The influence of initial values of three agents on evolutionary processes.

Based on the above research, we explored the convergence of the agents in the three-party game with different initial probabilities. Using the second set of values, while keeping the initial probabilities of the two variables in *x, y* and *z* fixed, we can infer how the initial probability of the third variable evolves over time, as shown in Figure 14.

As we can see from Figure 14, the initial probabilities of the three variables are assigned values of 0.2, 0.5, and 0.7, respectively. As the initial probabilities gradually increase, the convergence speed greatly accelerates, and all three agents quickly converge to their respective stable points, demonstrating that the initial probability significantly affects the speed of evolution.

## 5. Discussion

### 5.1. Policy Implications

Our research enriches the literature on green governance and greenwashing behaviour, and it has theoretical and practical implications for policymakers and high-pollution enterprises.

First, from a long-term perspective, government incentives and penalties associated with environmental regulation are still the most effective way to restrain environmental pollution and prompt high-pollution enterprises to strengthen their green governance. This is consistent with the conclusions of many works in the literature (e.g., [7,54,58,62]). A lack of government supervision decreases the seriousness and authenticity of enterprises’ environmental information efforts [31], and relevant government departments should establish joint supervision and audit mechanisms [7,63,64,65]. Therefore, state and local governments must improve the incentive-and-penalty mechanism, strictly punish greenwashing, more severely punish rent-seeking by enterprises, and adopt a ‘zero tolerance’ attitude to crack down on all violations [7].

Second, truthful reports from third-party certifiers are very important to both government and business. Due to the complexity of modern organisations, it is difficult to directly evaluate greenwashing behaviours [14]; therefore, verifiable reports from authoritative third parties can provide objective evaluations, which are effective in reducing greenwashing behaviours, especially in developing countries [66,67].

Third, green innovation is the fundamental way to resolve the conflictual relationship between economic growth and green governance [7,68]. Enterprises can only improve their core competitiveness through green innovation, which enables enterprises to reduce greenwashing to improve corporate social responsibility [69] and stabilise their development mode.

Fourth, supervision by third parties, such as NGOs, and the public [62,70], the media [62,70], especially social media [56,57,63,71], has a notable influence on green governance and the facilitation of green economic development [63]. Thus, it is necessary to increase media exposure, cultivate public attitudes towards green governance, and increase informatisation and other measures [63] that help to reduce the cost of local law enforcement, thereby improving overall supervision efficiency.

### 5.2. Limitations and Future Work

We recognise the existence of some limitations that may provide directions for future studies.

First, this paper only considers game tripartite green governance under asymmetric information and bounded rationality conditions, and it does not consider the influence of either the central government or the order of games. Therefore, it would be informative to consider the central government, the local government, the public, and the media as agents participating in the dynamic game process [28,29,70]. However, many studies only analyse social media as a perspective, and only a few scholars study the public [72] and social media [72] as the main body of the game. Therefore, future research in this direction will clarify the role of social media in the game in modern society. In addition, since the current study does not distinguish the influence of traditional media and social media on the game, future research can also focus on these two situations and clarify the similarities and differences in the role of traditional media and social media in the game.

Second, although we analyse the game of a three-party green governance system, we only analyse the participants’ interactions and strategic choices, which cannot provide a complete understanding of their impact. Factors such as subjective emotions [72] and the risk preference of the participants [73] largely affect the process and outcome of the game [72]. Future research can start from these factors and more comprehensively reflect the game subjects’ behaviour. Our analysis shows that the evolutionarily stable strategy (standard green governance, refusal to rent-seek, and loose supervision) entails loose supervision by the local government. A possible reason for this result is that the model ignores the influence of random factors. The introduction of a random process into the three-party game would further extend the results of this study [52,73,74,75]. Future research can address the relationship between corporate green innovation, enhanced social responsibility, and uncertainty in the outside world.

Finally, our theoretical models lack empirical data from a real-world case. In 2018, China implemented an environmental tax policy, which began to be levied in April 2022. This development provides a rich case for future scientific research. Therefore, a further research direction would be to improve on the design of the environmental tax mechanism based on a real-world case analysis. China’s success story can serve as a guide for other emerging countries facing transformational challenges and needing a positive attitude towards sustainability.

## 6. Conclusions

In the paper, we combine China’s green governance and the greenwashing behaviours of high-pollution enterprises to re-explore the game relationship and strategic choices among environmental regulations, high-pollution enterprises, and GCIs. We also explore the conditions under which local government environmental regulation is the most effective, and we draw the following conclusions.

First, stronger incentives and penalties by local governments facilitate the normative behaviour of high-pollution enterprises to strengthen green governance without greenwashing and incentivise third-party GCI to decline to rent-seek. This phenomenon may be due to the administrative advantages of local governments [17], and our result is consistent with those of many other researchers. However, increased incentives are detrimental to the government’s fulfilment of its own regulatory responsibilities because reliance on subsidies and incentives causes the local government to lack enthusiasm for active supervision.

Second, for the government’s incentive-and-penalty mechanism to be effective, it must ensure that each agent’s total incentives and penalties exceed its speculative gains, which prevents the green governance system from resulting in undesirable but stable strategies.

Finally, when a higher level of government holds a local regulatory body accountable, it encourages high-pollution enterprises to meet their green governance responsibilities without greenwashing and discourages the third-party GCI from rent-seeking, a result that has important practical significance. In addition, improving the operating profits of high-pollution enterprises and increasing the costs of rent-seeking are effective ways to discourage enterprises from greenwashing and failing to fulfil their environmental governance responsibilities.

Accordingly, we establish the connection between enterprises’ greenwashing behaviour, GCIs, and local government intervention.

## Figures and Tables

**Table 1:** Payoff matrix of high-pollution enterprises, local governments, and third-party GCIs.

Third-Party GCI			Local Government	
			Strict Supervision *z*	Loose Supervision 1 − *z*
High-pollution enterprises	No greenwashing behaviour *x*	Refusal to rent-seek *y*	$Q_{p}-C_{p1}+S_{P}$ $A_{e}+S_{e}$ $-C_{g}-S_{e}-S_{p}+D_{g}$	$Q_{p}-C_{p1}$ $A_{e}$ $D_{g}$
		Intention to rent-seek 1 − *y*	$Q_{p}-C_{p1}+S_{P}$ $A_{e}-C_{e}-F_{e}$ $-C_{g}-S_{p}+F_{e}+D_{g}$	$Q_{p}-C_{p1}$ $A_{e}-C_{e}$ $D_{g}$
	Greenwashing behaviour 1 − *x*	Refusal to rent-seek *y*	$-C_{p2}-C_{p}-F_{p}-T_{p}$ $A_{e}+S_{e}$ $-C_{g}+F_{p}+T_{p}-S_{e}$	$-C_{p2}-C_{p}-T_{p}$ $A_{e}$ 0
		Intention to rent-seek 1 − *y*	$Q_{p}-C_{p2}-C_{p}-R_{e}-F_{p}-T_{p}$ $A_{e}-C_{e}+R_{e}-F_{e}$ $-C_{g}+F_{p}+F_{e}+T_{p}-E_{g}$	$Q_{p}-C_{p2}-C_{p}-R_{e}-T_{p}$ $A_{e}-C_{e}+R_{e}$ $-E_{g}-F_{g}$

**Table 2:** Jacobian matrix eigenvalues.

Equilibrium Point	$\lambda_{1}$	$\lambda_{2}$	$\lambda_{3}$
M_1_ (0, 0, 0)	$C_{e}-R_{e}$	$R_{e}+C_{p}-C_{p1}+C_{p2}+T_{p}$	$F_{p}-C_{g}+F_{e}+T_{p}+F_{g}$
M_2_ (0, 1, 0)	$R_{e}-C_{e}$	$F_{p}-C_{g}-S_{e}$	$C_{p}-C_{p1}+C_{p2}+Q_{p}+T_{p}$
M_3_ (0, 0, 1)	$C_{e}-R_{e}+F_{e}+S_{e}$	$C_{g}-F_{p}-F_{e}-T_{p}-F_{g}$	$C_{p}-C_{p1}+C_{p2}+F_{p}+T_{p}+S_{p}+R_{e}$
M_4_ (0, 1, 1)	$C_{g}-F_{p}+S_{e}$	$R_{e}-C_{e}-F_{e}-S_{e}$	$C_{p}-C_{p1}+C_{p2}+F_{p}+S_{p}+Q_{p}+T_{p}$
M_5_ (1, 0, 0)	$C_{e}$	$F_{e}-C_{g}-S_{p}$	$C_{p1}-C_{p}-C_{p2}-R_{e}-T_{p}$
M_6_ (1, 1, 0)	$-C_{e}$	$-C_{g}-S_{p}-S_{e}$	$C_{p1}-C_{p}-C_{p2}-Q_{p}-T_{p}$
M_7_ (1, 0, 1)	$C_{e}+F_{e}+S_{e}$	$C_{g}-F_{e}+S_{p}$	$C_{p1}-C_{p}-C_{p2}-R_{e}-F_{p}-S_{p}-T_{p}$
M_8_ (1, 1, 1)	$C_{g}+S_{p}+S_{e}$	$-C_{e}-F_{e}-S_{e}$	$C_{p1}-C_{p}-C_{p2}-F_{p}-S_{p}-Q_{p}-T_{p}$

## Data Availability

Not applicable.
